# Supplementary material for: Bimodal Whole-Mount Imaging of Tendon Using Confocal Microscopy and X-ray Micro-Computed Tomography
Source: Biol Proced Online. 2020 Jul 1;22:13. doi: 10.1186/s12575-020-00126-4 (PMC7329428; doi:10.1186/s12575-020-00126-4)
Supplement: Supplementary file 1 — Additional file 1: Supplementary Figure 1. Schematic showing data collection and analysis workflows for confocal microscopy and x-ray microtomography. Supplementary Fig. 2: Representative 2D sections of equine superficial digital flexor and rat Achilles tendons, immunolabelled for LAMA4. Longitudinal sections were immunolabelled for LAMA4, using a primary antibody concentration of 1:200 in the equine SDFT (a) and 1:100 in the rat Achilles (c), detected with alexa-594 conjugated secondary antibody (1:500) and nuclei counterstained with DAPI. Negative controls, in which primary antibodies were omitted, were performed for both species (equine SDFT, b; rat Achilles, d). Scale bar = 100 μm. [file 12575_2020_126_MOESM1_ESM.docx]

# **SUPPLEMENTARY INFORMATION**

# **Supplementary Methods: 2D immunolabelling of equine SDFT and rat Achilles tendon to establish optimum antibody concentrations**

Longitudinal cryosections were cut from one SDFT (10 µm; 6 year old horse) and one rat Achilles tendon (12 µm; 13 week old female Wistar) that had been embedded in OCT and snap-frozen in n-hexane cooled on dry ice. Sections were adhered to glass slides and stored at -80 °C prior to immunolabelling. Sections were thawed, fixed with 4% PFA for 10 minutes and washed with TBS. Blocking conditions were consistent with those described for cleared samples used in the main study. Sections were incubated overnight at 4 °C with rabbit anti-LAMA4 primary antibody at dilutions ranging from 1:100 to 1:500 to establish optimum concentrations. Sections were washed with TBS, incubated with goat anti-rabbit Alexa Fluor® 594 secondary antibody (1:500, 2h, RT), coverslipped with Prolong Gold antifade mountant with DAPI (Invitrogen™ P36941) and cured overnight. Negative controls were included in which the primary antibody was omitted. Sections were imaged using a Leica TCS SP8 laser scanning confocal microscope a HC PL FLUOTAR 10x/0.32 dry objective lens.

A primary antibody concentration of 1:200 for the equine SDFT, and 1:100 for the rat Achilles provided optimal detection (Supp. Fig. 2). No non-specific labelling was detected in negative controls.

## **Supplementary Figures**

#
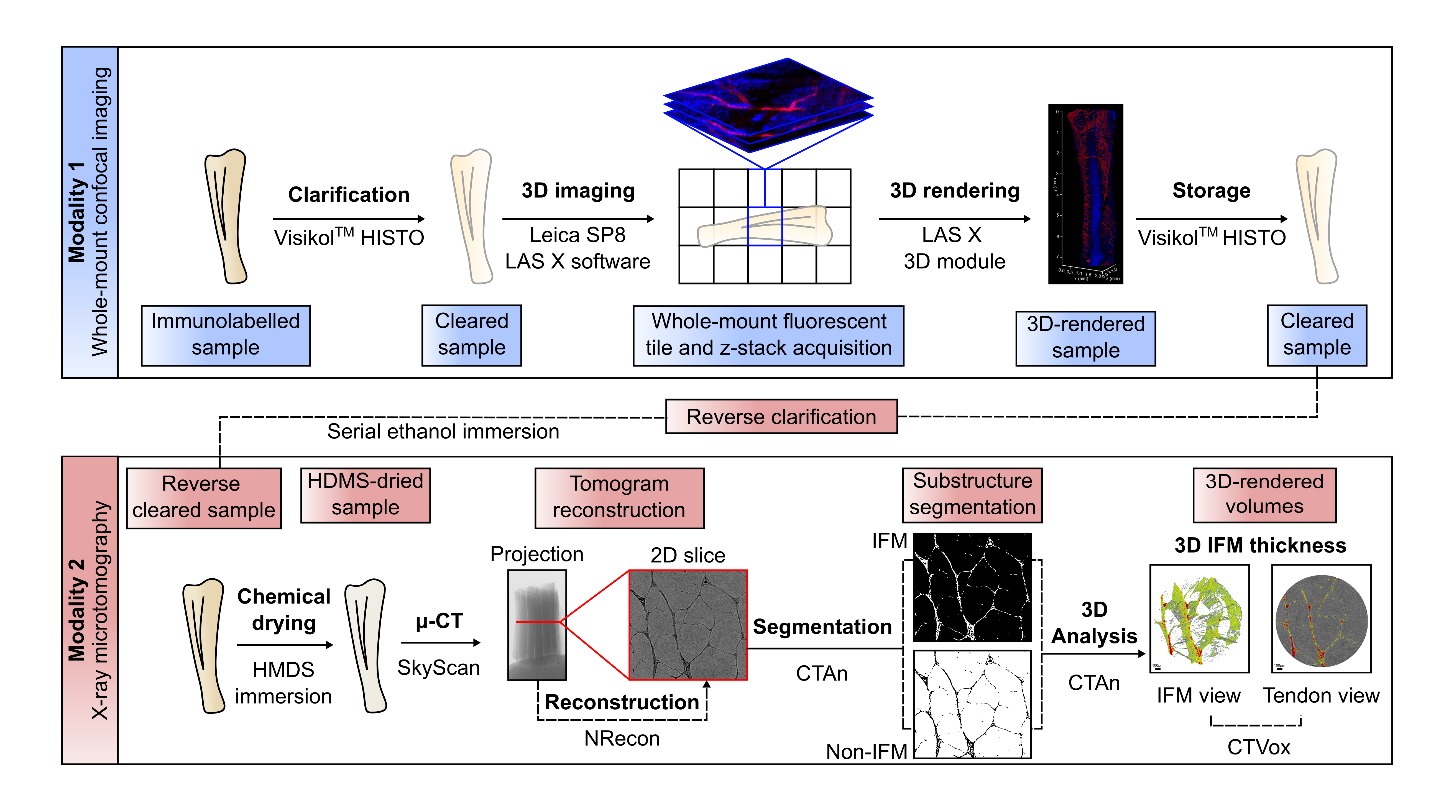


# Supplementary Figure 1. Schematic showing data collection and analysis workflows for confocal microscopy and x-ray microtomography.

##
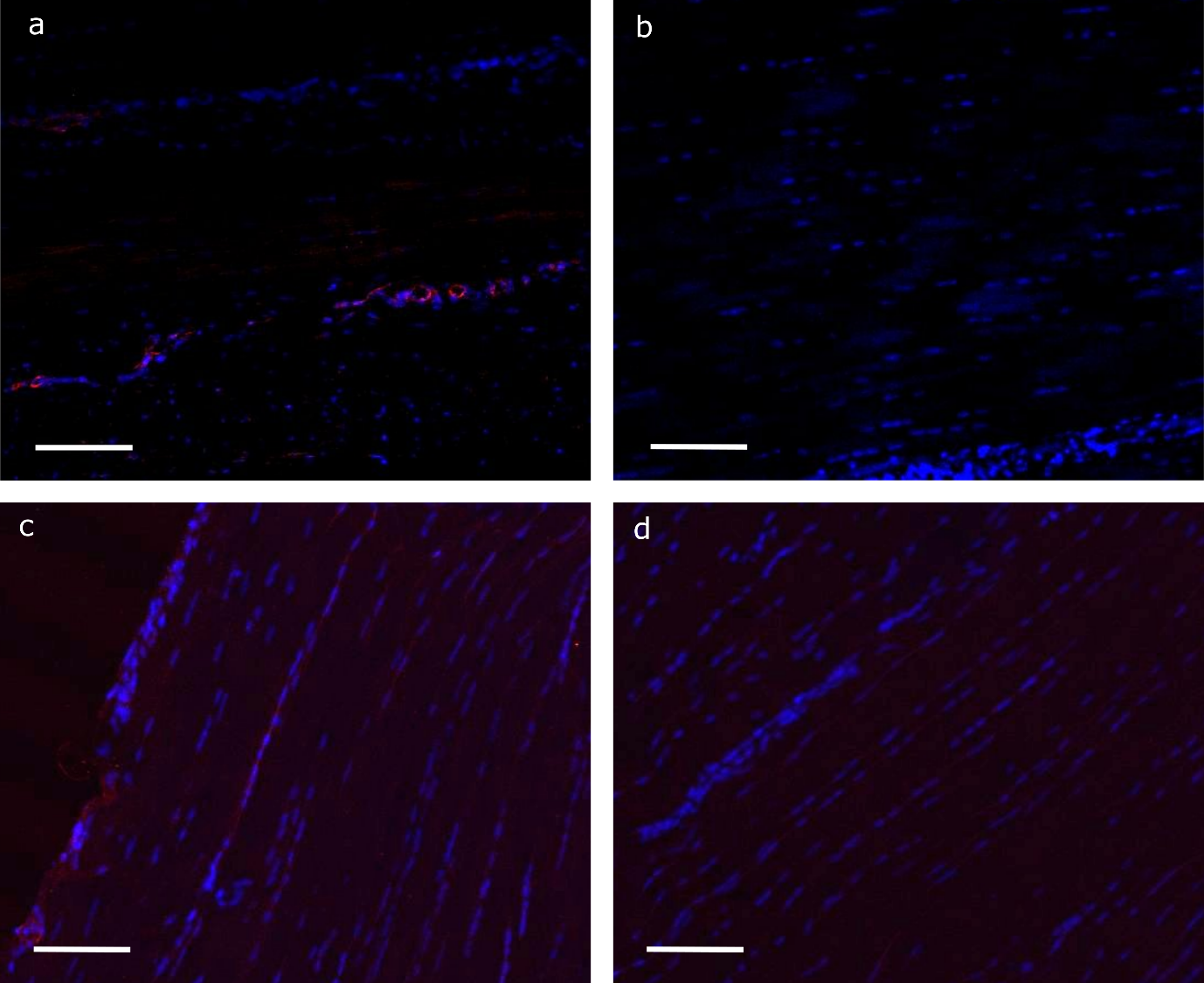
Supplementary Fig. 2: Representative 2D sections of equine superficial digital flexor and rat Achilles tendons, immunolabelled for LAMA4. Longitudinal sections were immunolabelled for LAMA4, using a primary antibody concentration of 1:200 in the equine SDFT (a) and 1:100 in the rat Achilles (c), detected with alexa-594 conjugated secondary antibody (1:500) and nuclei counterstained with DAPI. Negative controls, in which primary antibodies were omitted, were performed for both species (equine SDFT, b; rat Achilles, d). Scale bar = 100 µm.
